# Long-term persistence with rituximab in patients with rheumatoid arthritis

**DOI:** 10.1093/rheumatology/key036

**Published:** 2018-03-16

**Authors:** Alexander G S Oldroyd, Deborah P M Symmons, Jamie C Sergeant, Lianne Kearsley-Fleet, Kath Watson, Mark Lunt, Kimme L Hyrich, BSRBR-RA Contributors Group

**Affiliations:** 1 Arthritis Research UK Centre for Epidemiology, University of Manchester, Manchester, UK; 2 National Institute of Health Research Manchester Musculoskeletal Biomedical Research Centre, Manchester University NHS Foundation Trust, Manchester, UK; 3 Centre for Biostatistics, Manchester Academic Health Science Centre, Manchester, UK

**Keywords:** rheumatoid arthritis, epidemiology, DMARDs (biologic), pharmacology, biological therapies

## Abstract

**Objectives:**

To investigate the long term persistence of rituximab (RTX) in a large observational RA cohort, investigate persistence of RTX when used as a first or second line biologic DMARD (bDMARD), to characterize subsequent bDMARD treatment following RTX.

**Methods:**

Patients with RA starting treatment with RTX (MabThera) between 2008 and 2011 were recruited into the British Society for Rheumatology Biologics Register for RA. Duration of RTX treatment over the first 4 years after initiation was estimated via Kaplan-Meier estimates and the reason for discontinuation was ascertained. Subsequent bDMARD use following RTX discontinuation was characterised. Treatment survival in bDMARD-naïve (first line RTX use) and experienced (second line RTX use) cohorts was described.

**Results:**

One thousand six hundred and twenty-nine patients were recruited (1371 bDMARD-experienced and 258 bDMARD-naïve). Sixty percent of the whole cohort remained on RTX after 4 years. Ineffectiveness (46%) and death (24%) were the most common reason for RTX discontinuation. RTX discontinuation was associated with RF negativity for the bDMARD-experienced cohort. Of those that discontinued RTX, 46% initiated treatment with another bDMARD, with tocilizumab being the most common.

**Conclusion:**

This large study of patients initiating RTX treatment for severe RA found that 60% persisted with treatment after 4 years. This study also identified that RTX is tolerated well when used as a first or second line bDMARD.

Rheumatology key messages

- The majority of patients with RA treated with rituximab remain on treatment after four years.
- The three most common reasons for rituximab discontinuation in RA were ineffectiveness, death and adverse events.
- Rituximab is well tolerated when used as a first or second line biologic DMARD for RA.

## Introduction

Medication continuation rates are a good proxy measurement of treatment effectiveness and safety. Analysis of long term continuation of a number of biologic DMARDs (bDMARDs) in RA and identification of factors related to drug survival has previously highlighted important clinical trends and variations of response not initially identified in clinical trials [1–5]. In 2007 approval was given for the use of rituximab (RTX), an anti-CD20 mAb, in combination with MTX for the treatment of patients with severe active RA who have not adequately responded to TNF inhibitors (TNFi) [6]. The long term use of RTX treatment has not been investigated as widely as other bDMARD agents; this is primarily due to long term treatment data only now becoming available 10 years after the approval of RTX use. The distinct method and frequency of administration, wide variability of dosing intervals [7] and difficulty in capturing a discontinuation reason in research settings [8] has also limited research into long term continuation of RTX.

Only a small number of studies have investigated the long term continuation of RTX use and findings are variable [9–12]; further, the differing definitions of RTX discontinuation in previous studies hinders direct comparison. In part this relates to the challenges of defining treatment stop dates for an irregularly dosed therapy such as RTX. However, information on the long term continuation of RTX in RA may aid in managing patient and clinician expectations and the planning of future treatment options on an individual and population level.

Although use of RTX was approved for use in those who have not adequately responded to a TNFi agent, clinical practice sees a number of patients treated with RTX as their first bDMARD, usually due to the presence of comorbidities that preclude TNFi use. Results of long term continuation with RTX when used as a first line bDMARD in this distinct patient group has not been previously reported.

Therefore, using data from the British Society of Rheumatology Biologics Register for RA (BSRBR-RA), this study aims to characterize the long term use of RTX, identify reasons for treatment discontinuation, identify factors associated with treatment discontinuation and characterise subsequent bDMARD use in both bDMARD-naive and bDMARD-experienced cohorts.

## Methods

### Study population

Since 2001, the BSRBR-RA has collected prospective observational data on UK individuals with a rheumatologist diagnosis of RA starting treatment with bDMARD therapies with the aim of studying long-term clinical effectiveness and safety. Between 2008 and 2011, a cohort of patients commencing RTX was recruited. This included patients already registered with the BSRBR-RA for previous bDMARD therapy, primarily TNFi, who re-registered at the point of starting RTX as well as those new to the study. UK guidelines recommended RTX be prescribed, in combination with MTX, as two 1 gram infusions 2 weeks apart, with at least 6 months between doses, in patients who had failed treatment with TNFi [6]. MabThera (produced by Roche) was the only brand of RTX used during the study period.

For patients who were new to the BSRBR-RA, the consultant responsible for care was asked to complete a baseline questionnaire that collected data on the patient’s demographic characteristics, DAS-28 and its individual components (at the time RTX was started), comorbidities (from a tick list), previous and current conventional synthetic DMARDs (csDMARD) and prior bDMARD exposure, RF and smoking status. For patients already registered with the BSRBR-RA after receiving another bDMARD, the consultant was asked to complete a BSRBR-RA Short Baseline Form that collected data on DAS28 at the time RTX was started, current drug therapy and an updated list of comorbidities. The other patient demographic characteristics and details of prior bDMARDs were obtained from the patient’s initial registration with the BSRBR-RA. All patients were asked to complete a HAQ [13] at the point of starting RTX.

Follow-up data were captured from the hospital 6 monthly for 3 years and then annually thereafter. This data included details of further RTX therapy (dates of infusions) and/or discontinuation with reason: adverse event, ineffectiveness, remission, new bDMARD therapy, and adverse events. In addition, all patients were flagged with the cancer and death data linkage organisations in England, Wales, Scotland and Northern Ireland. In the case of death, the date and cause of death were forwarded to the study office. No data on lymphocyte levels were captured.

Ethical approval for the BSRBR-RA was granted by the North West Multicentre Research Ethics Committee in 2000. Written informed consent was gained at the time of registration into the BSRBR-RA cohort. No additional ethical approval or consent was required for this study.

### Data analysis

This analysis included all patients with RA registered at the point of starting RTX between February 2008 and December 2011 and included all follow-up data available to 30 November 2014. Patients were analysed as a whole group and also divided into two cohorts based on prior bDMARD exposure (bDMARD-experienced and bDMARD-naïve). Baseline data were compared between those with and without past bDMARD exposure using descriptive statistics.

All patients were considered to have continuous RTX use for 9 months (275 days) following each infusion/treatment course unless they started an alternative bDMARD, where the time of subsequent bDMARD initiation was defined as the stop date. For example, a patient who received a single course of RTX would be considered to have persisted with RTX for 9 months. This 9 month time frame was chosen to reflect the previously reported average time taken for B cell reconstitution to take place [14]. If a subsequent RTX infusion was received before the end of this 9 month period, a further 9 months would be added to the time on drug from the date of the subsequent infusion. As the length of B cell reconstitution is variable, for patients who received a further dose of RTX after the 9 month window, we did consider this continuous treatment. For this we allowed up to 15 months as long as an alternative bDMARD was not administered in the interval. Persistence with RTX was described using Kaplan-Meier survival analysis with estimates calculated at 1, 2, 3 and 4 years. Patients were censored at the time of death if it occurred within the 9 month period following last infusion. Each patient was recorded as having discontinued RTX treatment for one of the following reasons: ineffectiveness, adverse event, remission.

Baseline variables associated with RTX persistence were assessed using multivariable Cox-proportional hazards models; the models were developed via a stepwise backwards variable selection method. Investigated variables included demographic factors at baseline (gender, age, smoking status), RA-specific variables (DAS-28, disease duration, RF status, HAQ score), concurrent and past treatment (baseline glucocorticoid use, baseline MTX, number of past bDMARDs) and the total number of the following comorbidities as recorded on the baseline form: hypertension, physician reported interstitial lung disease (ILD), previous cancer, angina, previous myocardial infarction, previous stroke, epilepsy, asthma, chronic obstructive pulmonary disease, peptic ulcer disease, history of liver or renal impairment, multiple sclerosis, diabetes mellitus and depression. To avoid collinearity, two multivariable models were formed—one with the composite DAS28 score along with the other investigated variables, then a second model with the components of the DAS28 (tender joint count, swollen joint count, global health visual analogue score, ESR) along with the other investigated variables. Missing data were generated via multiple imputation [15]; this was carried out for all variables where missing data were present The statistical program R was used for all analysis [16].

The proportion of the cohort that initiated a subsequent bDMARD following RTX discontinuation at any point over the observed follow-up was calculated for both the bDMARD-naive and experienced cohorts.

## Results

### Study population

In total, 1629 patients were registered with the BSRBR-RA at the point of starting RTX, including 1371 (84%) bDMARD-experienced patients and 258 (16%) bDMARD-naive (Table 1). The median age of the population was 60 years [interquartile range (IQR): 52–68) and 76% were female. Only 68% of the cohort were reported to be RF positive (missing in 13%). Disease severity was high with a median baseline DAS28 score of 6.1 (IQR: 5.4–6.8) and median baseline HAQ score of 2.0 (IQR: 1.6–2.4). A majority of patients started RTX in combination only alongside MTX (64%), 2% only alongside LEF and 1% only alongside either only SSZ or HCQ. Twenty-three percent initiated RTX alongside MTX and one or more csDMARD. Ten percent of the cohort started RTX without a csDMARD. A majority of the bDMARD-experienced cohort started RTX after failing a single bDMARD (75%) and all but 15 patients had received a prior TNFi (the majority etanercept or adalimumab) (Table 2). Previous bDMARD data were not available for 93 (6.8%) of the bDMARD-experienced cohort. bDMARD-naïve patients were significantly older, more likely to be male, had shorter disease duration and lower HAQ scores than the bDMARD-experienced patients (Table 1). Although number of comorbidities was similar between each cohort, there were differences in the occurrence of individual comorbidities. In particular, a higher proportion of the bDMARD-naive cohort had either ILD or previous cancer. Ninety-five percent of the cohort received the recommended dose of RTX (two 1 gram infusions 14 days apart).

**Table 1:** Baseline characteristics of entire RTX cohort, bDMARD-experienced and bDMARD-naïve cohorts

Variable	Total cohort	bDMARD- naïve patients	bDMARD- experienced patients	*P*-value^a^	Missing data, *n* (%)
*N*	1629	258	1371		
Age at RTX initiation, median (IQR), years	60.8 (51.8–67.9)	64.2 (56.0–70.6)	60.2 (51.2–67.6)	<0.01	0
Female, *n* (%)	1243 (76)	174 (67)	1069 (78)	<0.01	0
Smoking status				<0.01	33 (2.0)
Never, *n* (%)	599 (37)	87 (34)	512 (37)		
Previously, *n* (%)	644 (40)	114 (44)	530 (39)		
Current, *n* (%)	354 (22)	56 (22)	298 (22)		
Comorbidities, *n* (%)				0.66	0
0	612 (38)	102 (40)	510 (37)		
1	518 (32)	77 (30)	441 (32)		
2	326 (10)	54 (21)	272 (20)		
3+	173 (11)	25 (9.7)	148 (11)		
ILD, *n* (%)	91 (5.6)	47 (18)	44 (3.2)	<0.01	0
Previous TB, *n* (%)	64 (3.9)	10 (3.9)	54 (3.9)	0.99	0
Previous cancer, *n* (%)	215 (13)	81 (31)	134 (9.8)	<0.01	0
Disease duration, median (IQR), years	12 (6–20)	10 (4–20)	13 (7–20)	<0.01	24 (1.5)
Swollen joint count,^b^ median (IQR)	8 (4–11)	8 (5–11)	8 (4–11)	0.19	25 (1.5)
Tender joint count,^b^ median (IQR)	13 (8–20)	13 (8–20)	13 (8–20)	0.74	28 (1.7)
Global health VAS, median (IQR)	71 (56–82)	70 (55–80)	72 (56–82)	0.42	35 (2.1)
ESR, median (IQR), mm/h	36 (20–62)	38 (20–60)	35 (20–63)	0.48	337 (21)
DAS28, median (IQR)	6.1 (5.4–6.8)	6.1 (5.5–6.7)	6.1 (5.4–6.9)	0.99	5 (0.3)
HAQ, median (IQR)	2.0 (1.6–2.4)	1.9 (1.5–2.3)	2.1 (1.6–2.4)	<0.01	215 (13)
Baseline oral glucocorticoid, *n* (%)	670 (41)	123 (48)	547 (40)	0.02	0
RF positive, *n* (%)	953 (68)	175 (68)	778 (57)	0.89	218 (13)
Concurrent MTX, *n* (%)	1043 (62)	137 (53)	906 (66)	0.01	0
Concurrent LEF, *n* (%)	129 (7.9)	27 (11)	102 (7.4)	0.12	0
No concurrent DMARD, *n* (%)	167 (10)	11 (4.3)	156 (11)	<0.01	0

a bDMARD-naïve *vs* bDMARD-experienced—continuous variables compared using Wilcoxon-signed rank test and proportions compared using Chi-squared.

b Out of the 28 joints as measured in the DAS28. bDMARD: biologic DMARD; RTX: rituximab; IQR: inter-quartile range; ILD: interstitial lung disease; TB: tuberculosis; VAS: visual analogue score; DAS28: Disease Activity Score 28.

**Table 2:** Number and names of previous bDMARDs in the bDMARD-experienced cohort

variable	*n* (%)
Number of previous bDMARDs
1	1029 (75)
2	222 (16)
3+	27 (2.0)
Unknown	93 (6.8)
Previous bDMARD	
Etanercept	652 (51)^a^
Adalimumab	558 (44)^a^
Infliximab	302 (24)^a^
Certolizumab	23 (1.8)^a^
Tocilizumab	5 (0.4)^a^
Anakinra	6 (0.5)^a^
Abatacept	3 (0.2)^a^
Ocrelizumab	3 (0.2)^a^

a Proportion of cohort that had been previously treated with bDMARD. bDMARD: biologic DMARD.

### Long-term persistence with RTX

**Fig. 1:**
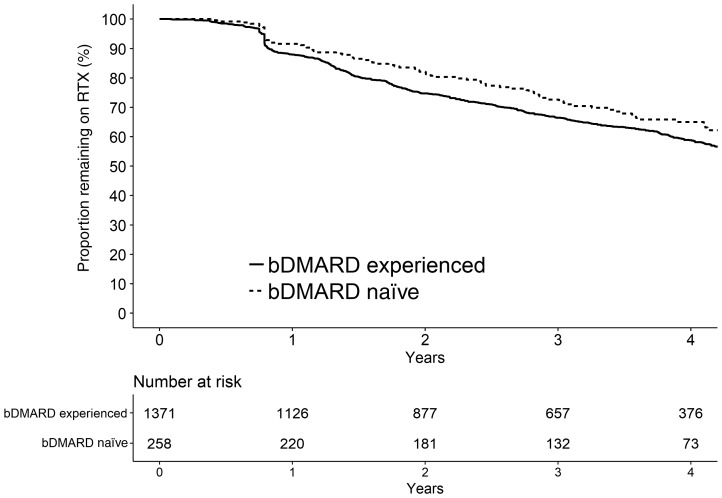
Kaplan-Meier plot of RTX continuation after 4 years in the bDMARD-experienced and naïve cohorts bDMARD: biologic DMARD; RTX: rituximab.

Seven thousand two hundred and eighty-six years of follow up data were available with a median follow up time of 4.5 years (IQR 3.6, 5.4). An estimated 60% (95% CI: 57%, 63%) of the whole cohort continued RTX after 4 years (Table 3). The RTX continuation estimate after 4 years was higher for the bDMARD-naive cohort, compared with the bDMARD-experienced cohort: 65% (95% CI: 59%, 72%) and 59% (95% CI: 56%, 62%), respectively (Fig. 1) although the general pattern of continuation was similar. Over the course of follow-up, the median number of RTX treatment courses was equal in each cohort: 3 (IQR 2–4). One quarter of patients only received a single course of RTX and then discontinued and almost 50% only received two courses.

**Table 3:** Proportion of patients remaining on RTX up to 4 years with discontinuation reason and subsequent biologic initiation

variable	Whole cohort, *n* = 1629	bDMARD-naïve patients, *n* = 258	bDMARD-experienced patients, *n* = 1371
No. of RTX courses (%)			
1	383 (24)	63 (24)	320 (23)
2	355 (22)	54 (21)	301 (22)
3+	891 (55)	141 (55)	750 (55)
Kaplan-Meier estimate after each year % (95% CI)			
1	89 (87, 90)	92 (88, 95)	88 (86, 90)
2	76 (74, 78)	82 (77, 87)	75 (72, 77)
3	67 (65, 70)	73 (67, 79)	66 (64, 69)
4	60 (57, 63)	65 (59, 72)	59 (56, 62)
Reported reason for discontinuation (presented as % of all stop reasons)			
Ineffectiveness, *n* (%)	260 (46)	30 (28)	230 (50)
Death, *n* (%)	137 (24)	36 (33)	101 (22)
Adverse event, *n* (%)	95 (17)	14 (13)	81 (18)
Remission, *n* (%)	7 (1)	3 (3)	4 (1)
Unknown, *n* (%)	68 (12)	25 (23)	43 (9)

bDMARD: biologic DMARD; RTX: rituximab.

### Reasons for RTX discontinuation

For the whole cohort, the most common reason for RTX discontinuation was ineffectiveness (46%). One hundred and thirty-seven patients died following RTX and this constituted 24% of the total reasons for discontinuation. Death was the most common reason for RTX discontinuation in the bDMARD-naïve cohort (33% of reasons for RTX discontinuation) while ineffectiveness was the most common reason in the bDMARD-experienced cohort (50% of reasons for RTX discontinuation). A higher proportion of the bDMARD-naïve cohort died, compared with the bDMARD-experienced cohort: 14 *vs* 7.4%, respectively (χ^2^*P* ⩽ 0.01). Remission constituted only 1.2% of the discontinuation reasons in the whole cohort. The reason for RTX discontinuation was not identified in 12% of the whole cohort.

### Factors associated with RTX discontinuation

For the whole cohort as well as a model limited to bDMARD-experienced patients, multivariable analysis revealed that RTX discontinuation was only associated with RF negativity [whole cohort hazard ratio (HR) = 0.74 (95% CI: 0.64, 0.87)]. Previous bDMARD use was not significantly associated with RTX discontinuation. A model limited to bDMARD-naïve patients did not identify any independent variables associated with RTX discontinuation although the sample size was small.

### Subsequent bDMARD use

Of those that discontinued RTX within 4 years due to reasons other than death, 263 (61%) subsequently initiated treatment with a different bDMARD (Table 4). A significantly higher proportion of the bDMARD-experienced cohort was treated with a subsequent bDMARD, compared with the bDMARD-naïve group (χ^2^*P* = 0.01). The median time to first subsequent bDMARD following the last RTX dose was 1.5 years (IQR: 0.9–2.5) for the whole cohort, 2 years (IQR: 1.2–3.1) for the bDMARD-naïve cohort and 1.5 years (IQR: 0.9–2.5) for the bDMARD-experienced cohort. Tocilizumab was the most commonly used subsequent bDMARD in each cohort.

**Table 4:** Profile of subsequent biologic drugs used following RTX discontinuation

bDMARD,^a^*n* (%)	Whole cohort, *n* = 1629	bDMARD-naïve cohort, *n* = 258	bDMARD-experienced cohort, *n* = 1371
Any	263 (61^b^)	29 (40^b^)	234 (65^b^)
Tocilizumab	145 (55)	12 (41)	133 (57)
Abatacept	30 (11)	2 (6.9)	28 (12)
Any TNFi	88 (34)	15 (52)	73 (31)
Etanercept	31 (12)	9 (31)	22 (9.4)
Adalimumab	25 (9.5)	3 (10)	22 (9.4)
Certolizumab	15 (5.7)	1 (3.4)	14 (6.0)
Infliximab	14 (5.3)	2 (6.9)	12 (5.1)
Golimumab	3 (1.1)	0 (0.0)	3 (1.3)

a Percentages represent proportion of those that initiated a subsequent bDMARD, unless stated otherwise.

b Proportion of those that had discontinued RTX within 4 years, excluding deceased patients. bDMARD: biologic DMARD.

## Discussion

This study has described long term use of RTX in a large cohort of patients with RA in routine clinical practice, demonstrating that 60% of patients continued RTX after 4 years and that continuation was higher for patients who were RF positive. Reasons for RTX discontinuation and subsequent bDMARD use were also characterized, with the most common reason for stopping being ineffectiveness and secondly death of the patient, consistent with previous reports [9, 17]. One quarter of patients who commenced RTX only received a single course of treatment and almost one half only received two courses, suggesting decisions to not persist with RTX are made early in the course of treatment.

Fifty-nine percent of the bDMARD-experienced cohort continued RTX after 4 years, indicating a high degree of effectiveness and tolerability as a second line bDMARD. Our continuation rates are similar to reported figures in other studies 85% after 1 year by Harrold *et al.* [18], 70% continuation after 3 years by Richter *et al.* [11] and 50% after 4 years by De Keyser *et al.* [9].

Our study’s continuation rates indicate that RTX, as a second line bDMARD, appears to be better tolerated than second line TNFi agents, as reported in other studies. A study of 235 Danish patients with RA revealed that after 1 year only 65% of the cohort continued a second line bDMARD [1], which is lower than the 89% continuing RTX after 1 year in our study. Further, a study by Gomez-Reino *et al.* [19] estimated that after 2 years, 60% of patients continued a second line TNFi for treatment of chronic arthritis (including RA, AS and PsA), lower than the 76% continuing RTX after 2 years in our study. It is therefore plausible that treatment with RTX may be a more appropriate option following failure of the first TNFi in selected patients, rather than treatment with an alternative TNFi, although understanding the reason for discontinuation may alter this.

The only baseline variable found to be associated with long-term RTX continuation was RF status. The relationship between RF status and response/continuation of RTX has been identified in previous work within the BSRBR-RA cohort, where greater response to RTX after 6 months was seen in those who were RF positive [20]. The finding that RF status was the only baseline variable associated with continuation indicates that prediction of an individual patient’s chance of continuing RTX long term may be challenging.

This study also described the treatment persistence in patients who are bDMARD naïve at the time of starting RTX. Although not licensed for this indication, there were 258 patients enrolled in our study receiving RTX as a first line bDMARD. Many of these patients had a relative contraindication for TNFi, such as prior cancer or ILD, which may explain why they had not received a TNFi first. Within this cohort, 66% were still receiving RTX after 4 years, supporting that this may be an effective treatment option for patients where TNFi cannot be given.

A notable finding of this study was the higher proportion of the bDMARD-naïve cohort that had died after 4 years, compared with the bDMARD-experienced cohort: 14 *vs* 7%, respectively. This difference may reflect the older age and higher comorbidity burden in the bDMARD-naïve cohort, the latter which may have been the reason RTX was chosen as a first line bDMARD.

Tocilizumab was the most common subsequent bDMARD used in our cohort. The predominant use of this agent following RTX discontinuation has been reported previously [21] and is likely due to many factors, including the dates of availability of the various treatment options and also the fact that many patients had already failed more than one TNFi prior to starting RTX. That the bDMARD naïve patients were also less likely to receive a TNFi following RTX also supports that this group likely continued to have contraindications to TNFi.

### Strengths

The large size of this study cohort and the availability of long-term follow up data are major strengths; the inclusion of a bDMARD naïve cohort also for the first time describes outcomes in patients within this treatment history. A further strength is the accurate capture of all cancer diagnoses and deaths through linkage of the cohort to corresponding national registers.

### Limitations

The level of missing data is a weakness of this study; for example, RF status was missing in 13% of the cohort. The absence of data on anti-CCP positivity prohibited investigation into its relationship with RTX discontinuation. Ascertaining an RTX discontinuation reason presented a challenge as only one reason for RTX discontinuation was recorded, even though the decision to discontinue treatment may have been multifactorial.

## Conclusions

This study of a large cohort of patients initiating RTX treatment for severe RA highlighted that 60% persisted with treatment after 4 years. This study also identified that RTX is also tolerated well when used as first line bDMARD. These findings should be of value to clinicians when selecting a first or second line bDMARD for patients with RA.

## References

[1] Hjardem E, Østergaard M, Pødenphant J Do rheumatoid arthritis patients in clinical practice benefit from switching from infliximab to a second tumor necrosis factor alpha inhibitor? Ann Rheum Dis 2007;66:1184–9. 17389656 10.1136/ard.2006.054742 PMC1955158

[2] De Keyser F, De Kock J, Leroi H Ten-year followup of infliximab therapy in rheumatoid arthritis patients with severe, longstanding refractory disease: a cohort study. J Rheumatol 2014;41:1276–81. 24882838 10.3899/jrheum.131270

[3] Kristensen LE, Saxne T, Nilsson J-A, Geborek P. Impact of concomitant DMARD therapy on adherence to treatment with etanercept and infliximab in rheumatoid arthritis. Results from a six-year observational study in southern Sweden. Arthritis Res Ther 2006;8:R174. 17121678 10.1186/ar2084 PMC1794519

[4] Listing J, Strangfeld A, Rau R Clinical and functional remission: even though biologics are superior to conventional DMARDs overall success rates remain low–results from RABBIT, the German biologics register. Arthritis Res Ther 2006;8:R66. 16600016 10.1186/ar1933 PMC1526636

[5] Strangfeld A, Hierse F, Kekow J Comparative effectiveness of tumour necrosis factor inhibitors in combination with either methotrexate or leflunomide. Ann Rheum Dis 2009;68:1856–62. 19126559 10.1136/ard.2008.098467

[6] National Institute for Health and Care Excellence. Adalimumab, etanercept, infliximab, rituximab and abatacept for the treatment of rheumatoid arthritis after the failure of a TNF inhibitor . NICE guideline 2010; (TA195).

[7] Keystone E, Fleischmann R, Emery P Safety and efficacy of additional courses of rituximab in patients with active rheumatoid arthritis: an open-label extension analysis. Arthritis Rheum 2007;56:3896–908. 18050221 10.1002/art.23059

[8] Johnston SS, McMorrow D, Farr AM, Juneau P, Ogale S. Comparison of biologic disease-modifying antirheumatic drug therapy persistence between biologics among rheumatoid arthritis patients switching from another biologic. Rheumatol Ther 2015;2:59–71. 27747492 10.1007/s40744-014-0006-3 PMC4883249

[9] De Keyser F, Hoffman I, Durez P, Kaiser M-J, Westhovens R. MIRA Study Group. Longterm followup of rituximab therapy in patients with rheumatoid arthritis: results from the Belgian MabThera in Rheumatoid Arthritis registry. J Rheumatol 2014;41:1761–5. 25128506 10.3899/jrheum.131279

[10] Nicholls D, Zochling J, Boers A A retrospective chart review of the use of rituximab for the treatment of rheumatoid arthritis in Australian rheumatology practice. Int J Rheum Dis 2014;17:755–61. 24131467 10.1111/1756-185X.12164

[11] Richter A, Strangfeld A, Herzer P Sustainability of rituximab therapy in different treatment strategies: results of a 3-year followup of a German Biologics Register. Arthritis Care Res 2014;66:1627–33. 10.1002/acr.22327 PMC4282041 24664818

[12] Wei W, Knapp K, Wang L Treatment persistence and clinical outcomes of tumor necrosis factor inhibitor cycling or switching to a new mechanism of action therapy: real-world observational study of rheumatoid arthritis patients in the United States with prior tumor necrosis factor inhibitor therapy. Adv Ther 2017;34:1936–52. 28674959 10.1007/s12325-017-0578-8 PMC5565674

[13] Kirwan JR, Reeback JS. Stanford Health Assessment Questionnaire modified to assess disability in British patients with rheumatoid arthritis. Br J Rheumatol 1986;25:206–9. 3708236 10.1093/rheumatology/25.2.206

[14] Teng YK, Huizinga TW, van Laar JM. Targeted therapies in rheumatoid arthritis: focus on rituximab. Biologics 2007;1:325–33. 19707303 PMC2721296

[15] Buuren S. v, Groothuis-Oudshoorn K. Mice: multivariate imputation by chained equations in *R*. J Stat Softw 2011;45:1–67.

[16] R Core Team. R: A Language and Environment for Statistical Computing. 2014 R Foundation for Statistical Computing, Vienna, Austria.

[17] Soliman MM, Hyrich KL, Lunt M Rituximab or a second anti-TNF therapy for rheumatoid arthritis patients who have failed their first anti-TNF? Comparative analysis from the British Society for Rheumatology Biologics Register. Arthritis Care Res 2012;64. 10.1002/acr.21663 PMC3492906 22422731

[18] Harrold LR, Reed GW, Magner R Comparative effectiveness and safety of rituximab versus subsequent anti–tumor necrosis factor therapy in patients with rheumatoid arthritis with prior exposure to anti–tumor necrosis factor therapies in the United States Corrona registry. Arthritis Res Ther 2015;17:256. 26382589 10.1186/s13075-015-0776-1 PMC4574482

[19] Gomez-Reino JJ, Carmona L. BIOBADASER Group. Switching TNF antagonists in patients with chronic arthritis: an observational study of 488 patients over a four-year period. Arthritis Res Ther 2006;8:R29. 16507128 10.1186/ar1881 PMC1526564

[20] Soliman MM, Hyrich KL, Lunt M Effectiveness of rituximab in patients with rheumatoid arthritis: observational study from the British Society for Rheumatology Biologics Register. J Rheumatol 2012;39:240–6. 22174201 10.3899/jrheum.110610

[21] Valleala H, Korpela M, Hienonen-Kempas T Long-term real-life experience with rituximab in adult Finnish patients with rheumatoid arthritis refractory or with contraindication to anti–tumor necrosis factor drugs. J Clin Rheumatol 2015;21:24–30. 25539430 10.1097/RHU.0000000000000166

